# Kakila database: Towards a FAIR community approved database of cetacean presence in the waters of the Guadeloupe Archipelago, based on citizen science

**DOI:** 10.3897/BDJ.9.e69022

**Published:** 2021-07-22

**Authors:** Lorraine Coché, Elie Arnaud, Laurent Bouveret, Romain David, Eric Foulquier, Nadège Gandilhon, Etienne Jeannesson, Yvan Le Bras, Emilie Lerigoleur, Pascal Jean Lopez, Bénédicte Madon, Julien Sananikone, Maxime Sèbe, Iwan Le Berre, Jean-Luc Jung

**Affiliations:** 1 LETG, IUEM UBO, Brest, France LETG, IUEM UBO Brest France; 2 PNDB (Pôle national de données de Biodiversité), UMS 2006 PatriNat, Concarneau, France PNDB (Pôle national de données de Biodiversité), UMS 2006 PatriNat Concarneau France; 3 OMMAG, Port Louis, France OMMAG Port Louis France; 4 ERINHA (European Research Infrastructure on Highly Pathogenic Agents), Bruxelles, Belgium ERINHA (European Research Infrastructure on Highly Pathogenic Agents) Bruxelles Belgium; 5 BREACH NPO, Ponteilla, France BREACH NPO Ponteilla France; 6 Sanctuaire Agoa, Les trois Ilets, France Sanctuaire Agoa Les trois Ilets France; 7 UMR 5602 CNRS GEODE, Toulouse, France UMR 5602 CNRS GEODE Toulouse France; 8 Observatoire Hommes-Milieux Littoral Caraïbe, Pointe-à-Pitre, France Observatoire Hommes-Milieux Littoral Caraïbe Pointe-à-Pitre France; 9 Laboratoire BOREA, MNHN/CNRS/SU/IRD/UCN/UA, Paris, France Laboratoire BOREA, MNHN/CNRS/SU/IRD/UCN/UA Paris France; 10 AMURE, IUEM UBO, Brest, France AMURE, IUEM UBO Brest France; 11 PNDB (Pôle national de données de Biodiversité), UMS 2006 PatriNat, Paris, France PNDB (Pôle national de données de Biodiversité), UMS 2006 PatriNat Paris France; 12 Aix Marseille Univ., Universite de Toulon, CNRS, IRD, MIO UM 110, Marseille, France Aix Marseille Univ., Universite de Toulon, CNRS, IRD, MIO UM 110 Marseille France; 13 Centre de Recherche en Gestion, École Polytechnique, Paris, Bâtiment Ensta, Palaiseau, France Centre de Recherche en Gestion, École Polytechnique, Paris, Bâtiment Ensta Palaiseau France; 14 Institut de Systématique, Evolution, Biodiversité (ISYEB), Muséum national d'Histoire naturelle, CNRS, Sorbonne Université, EPHE, Université des Antilles, Brest, France Institut de Systématique, Evolution, Biodiversité (ISYEB), Muséum national d'Histoire naturelle, CNRS, Sorbonne Université, EPHE, Université des Antilles Brest France; 15 Université de Brest, Brest, France Université de Brest Brest France

**Keywords:** cetaceans, citizen science, observation, database, FAIR, French West Indies

## Abstract

**Background:**

In the French West Indies, more than 20 species of cetaceans have been observed over the last decades. The recognition of this hotspot of biodiversity of marine mammals, observed in the French Exclusive Economic Zone of the West Indies, motivated the French government to create in 2010 a marine protected area (MPA) dedicated to the conservation of marine mammals: the Agoa Sanctuary. Threats that cetacean populations face are multiple, but well-documented. Cetacean conservation can only be achieved if relevant and reliable data are available, starting by occurrence data. In the Guadeloupe Archipelago and in addition to some data collected by the Agoa Sanctuary, occurrence data are mainly available through the contribution of citizen science and of local stakeholders (i.e. non-profit organisations (NPO) and whale-watchers). However, no observation network has been coordinated and no standards exist for cetacean presence data collection and management.

**New information:**

In recent years, several whale watchers and NPOs regularly collected cetacean observation data around the Guadeloupe Archipelago. Our objective was to gather datasets from three Guadeloupean whale watchers, two NPOs and the Agoa Sanctuary, that agreed to share their data. These heterogeneous data went through a careful process of curation and standardisation in order to create a new extended database, using a newly-designed metadata set. This aggregated dataset contains a total of 4,704 records of 21 species collected in the Guadeloupe Archipelago from 2000 to 2019. The database was called Kakila ("who is there?" in Guadeloupean Creole). The Kakila database was developed following the FAIR principles with the ultimate objective of ensuring sustainability. All these data were transferred into the PNDB repository (Pöle National de Données de Biodiversité, Biodiversity French Data Hub, https://www.pndb.fr).

In the Agoa Sanctuary and surrounding waters, marine mammals have to interact with increasing anthropogenic pressure from growing human activities. In this context, the Kakila database fulfils the need for an organised system to structure marine mammal occurrences collected by multiple local stakeholders with a common objective: contribute to the knowledge and conservation of cetaceans living in the French Antilles waters. Much needed data analysis will enable us to identify high cetacean presence areas, to document the presence of rarer species and to determine areas of possible negative interactions with anthropogenic activities.

## Introduction

Roughly 40% of the world’s human population live within 100 km of a coast*^1^ and its growth is putting an unprecedented pressure on coastal and marine ecosystems and their organisms (Burke et al. 2001, Halpern et al. 2015). In particular, shipping now accounts for more than 90% of global trade, it is constantly increasing, resulting in an expanding consumption of coastal land and a continuous increase in the intensity of maritime traffic and the size of its vessels (Sèbe 2020, UNCTAD 2018, Walker et al. 2019). If we want to mitigate the consequences of these changes, it is essential to monitor our impacts on the oceans and their ecosystems and to collect relevant data for this purpose. In particular, the monitoring of marine mammal populations may contribute to a better understanding of the interactions between the growing pressure of human maritime activities and their environment. Indeed, cetacean populations are considered as sentinel and umbrella species, because their presence testifies to the functional importance of the marine realm for the conservation of the environment (Hooker and Gerber 2004, Jung and Madon In press). However, scientific surveys generally require costly human and financial resources to implement the sampling protocols that are required to estimate robust relative abundance and density of cetacean species at sufficiently fine spatial and temporal scales (Laran et al. 2017, Pennino et al. 2017, Rone et al. 2016). To address these constraints, complementary methods are needed to extend spatial and temporal coverage and to collect additional data. In this context, citizen-science, in which part of the research is conducted by volunteer non-professional scientists, represents a highly relevant alternative to scientific surveys to acquire additional data at lower cost and often at larger spatial and temporal scales. Thus, in many situations and places where scientific data cannot be collected, data provided by citizens is an invaluable source of information. For marine mammals, relevant examples are, for instance, the Monicet platform in the Azores (http://www.monicet.net), the Flukebook catalogue (Levenson et al. 2015), the Gotham Whale project near New York City (https://gothamwhale.org), the Intercet platform in the northern Tyrrhenian Sea (http://www.intercet.it), the network Obsenmer in some places of French waters (https://www.obsenmer.org) or the recently published data obtained in Kenya (Mwango’mbe et al. 2021). Although the data acquired by citizen science can be opportunistic and ultimately heterogeneous, it has been shown that it can reveal the same trends as those highlighted by data obtained through scientific surveys (Harvey et al. 2018, Jung et al. 2009, Stelle 2017, Van Strien et al. 2013).

The Guadeloupe Archipelago is a hotspot of marine biodiversity where understanding the interactions between cetaceans and human activities is essential. It has also led the French government to create a marine protected area dedicated to marine mammals within the French Exclusive Economic Zone of the West Indies: the Agoa Sanctuary. However, adequate cetacean conservation can only be achieved if relevant and reliable data are available. In the Guadeloupe Archipelago, besides a PhD thesis (Gandilhon 2012) and few scientific observation surveys (Boisseau et al. 2006, Laran et al. 2019, Van Canneyt et al. 2009), occurrence data are only available thanks to the contribution of dedicated local citizen-science stakeholders (i.e. NPOs and whale-watching companies). These data are highly valuable, often made by experienced observers able to accurately distinguish between species and some of them were used for scientific targeted studies (Heenehan et al. 2019, Kennedy et al. 2014, Stevick et al. 2016, Stevick et al. 2018). By their very heterogeneous nature, citizen science data are challenging to analyse (Van Strien et al. 2013). That is why it makes sense to integrate them into a database complying with the FAIR principles (Wilkinson et al. 2016) using a step-by-step community approach (David et al. 2020) and a pragmatic method taking into account the constraints of the stakeholders (Jacob et al. 2020). All of this is with the aim of promoting their sharing and dissemination within the scientific community interested in marine mammals and marine spatial planning.

**Figure 1.**
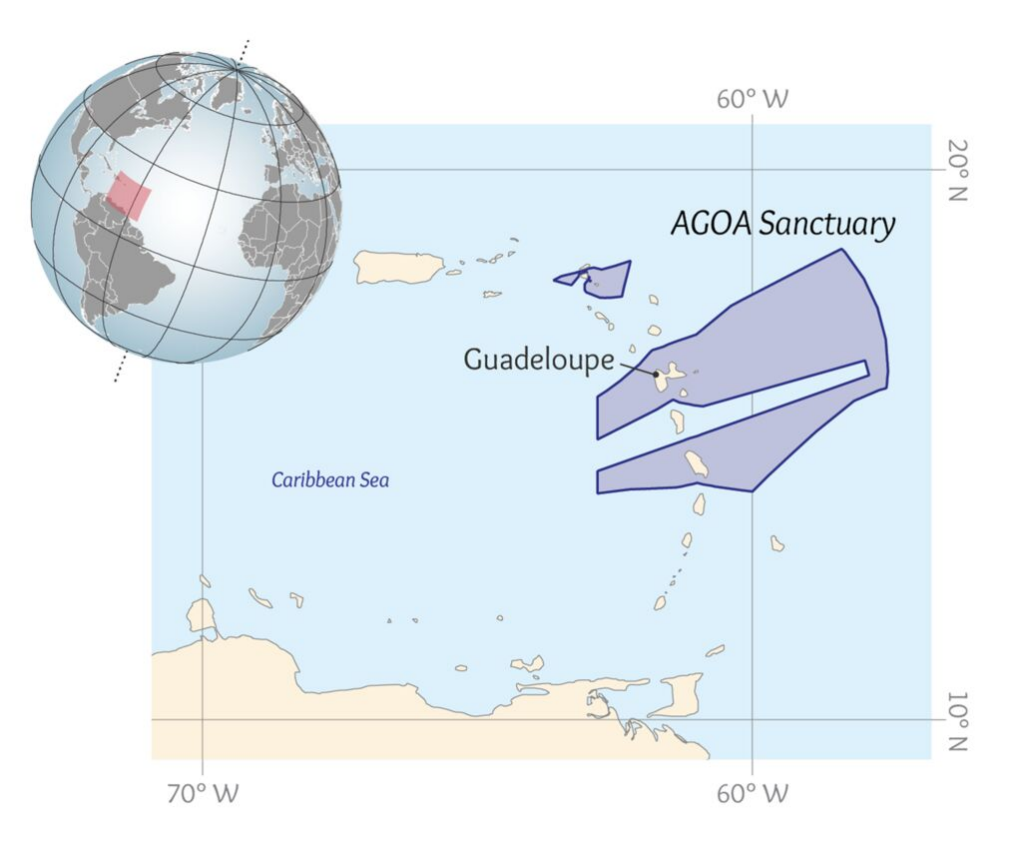
Area of study. Perimeter of the the Agoa Sanctuary, which corresponds to the French Economic Zone in the West Indies and localisation of the Guadeloupe Archipelago (data sources: map base, http://www.caribbeanmarineatlas.net; Agoa protection zone, https://inpn.mnhn.fr).

This data paper presents the process of structuring heterogeneous multi-source data in order to build a robust and standardised database of cetacean observations around the Guadeloupe Archipelago (Fig. 1). Observations collected over several years by local NPOs or whale watchers (Figs 2, 3) have been integrated into a database named "Kakila" (namely “who is there” in the Guadeloupean Creole language). The data processing steps, their curation protocol, quality assurance processes and the methods and tools that enable the long-term integrity and comprehension of data are presented. The Kakila database has been added into the PNDB repository (Pôle National de Données de Biodiversité, Biodiversity French Data Hub, https://www.pndb.fr).

## Project description

### Design description

**The FAIRification process of the Kakila database (Table 1).** The key goal of our project was to group heterogeneous, but scientifically significant datasets of cetacean observations in the Guadeloupe Archipelago into a single database and to make it open access. To achieve this goal, we followed the FAIR guiding principles (Wilkinson et al. 2016). According to the European and International Open Science dynamic, the French National Plan for Open Science (Ministère de l’Enseignement Supérieur, de la Recherche et de l’Innovation 2018) aims to ensure that data produced by government-funded research in France are gradually structured to comply with the FAIR Data Principles (Findable, Accessible, Interoperable and Reusable) (Wilkinson et al. 2016). We also followed the "as open as possible, as closed as necessary" principle of the H2020 Programme Guidelines on FAIR Data (Landi et al. 2020), by deleting, from this shared version, the observer names to avoid the dissemination of personal data. As a consequence, the chosen strategy for the FAIRification process mainly used the recommendations of the Sharing Rewards and Credit (SHARC) IG (Interest Group of the Research Data Alliance), particularly the FAIR assessment decision-tree criteria and lessons learned for the gradual implementation of FAIR criteria (David et al. 2020).

**Deposit to national and international aggregators.** In order to allow a wide dissemination and to improve its accessibility, the Kakila database content has been deposited in the PNDB (Pôle National de Données de Biodiversité, Biodiversity French Data Hub, https://www.pndb.fr) infrastructure data repository. In accordance with DataOne network guidelines, data were structured using rich metadata thanks to the use of the Ecological Metadata Language (EML) v.2.2.0 (Jones et al. 2019) and a data package has been created preliminary to deposition. Metadata addition and data package creation were made through the MetaShARK v.1.3 Shiny app (Arnaud et al. 2020) and the use of EML Assembly Line R Package (EMLassemblyline 2019). The resulting data package has been then submitted to the PNDB metadata catalogue (Jones et al. 2020) accessible at https://data.pndb.fr/.

## Sampling methods

### Study extent

The data were collected around the Guadeloupe Archipelago (Fig. 1) by seven different stakeholders starting in 2000. One NPO collected marine mammal observations during daily trips: OMMAG (Observatoire des Mammifères Marins de l'Archipel Guadeloupéen or "Observatory of marine mammals of the Guadeloupe Archipelago"). Another NPO, BREACH, performed line transects and made available for this study the observation data (Gandilhon 2012). Two datasets were provided by the Agoa Sanctuary, compiling observations made between 2012 and 2016. Finally, several professional whale-watchers (Guadeloupe Evasion Découverte located in Deshaie, Cétacés Caraïbes located in Bouillante and Aventures Marines located in Gourbeyre) also provided access to the data they recorded during daily tours. We also integrated into the Kakila database open-access data coming from observation surveys conducted by the IFAW (International Fund for Animal Welfare) in 1995, 1996, 2000 and 2006 (Boisseau et al. 2006).

### Sampling description

Sampling consisted, in a first phase, in conducting a preliminary survey of the different NPOs and professional whale-watchers known to record cetacean observation data around the Guadeloupean Archipelago and whose expertise was previously recognised: for example, co-authorship of scientific publications (Barragán-Barrera et al. 2019, Stevick et al. 2016, Heenehan et al. 2019, Stevick et al. 2018), participation to a PhD (Gandilhon 2012) and book publication (e.g. Mon école ma baleine 2019). We established contacts to collaborate and to agree on the terms of use and fair sharing of the data into a common database. Following this first survey, an informal invitation to open and contribute their dataset was sent to each organisation. All agreed to share and open the data once the aggregated database would be finalised.

**Data description**: the data consisted in marine mammal species observations collected during daily-boat excursions related to citizen science data acquisition or related to tourism (whale watching) (Figs 2, 3). Observations were enriched with various environmental information (visibility, sea state ...), detailed in the Dataset "sortie" (Trip) (Table 3). Geolocation coordinates were often provided. A specific level of expertise was assigned to each observer (i.e. beginner, intermediate, expert levels) in order to attest the robustness of the observation. The observation data were collected in French.

### Quality control

An effort to centralise and harmonise siloed data was made by controlling the join keys (eg. "code_observation", "code_sortie" etc.) between linked tables using dynamic pivot tables. Content quality controls were also used, such as a controlled dropdown menu for many fields that avoid potential input errors. Geolocations, often transformed into decimal degrees, were verified using the Geographic Information System QGIS 3.10 (long-term release) software.

In addition, data were checked for errors: 10% of the entries were randomly selected and checked by two persons. One person carried out the random draw from the “observation” table and the other operator checked the selected lines in the database against the original datasets provided by the data owners. The data entry was invalidated if it contained an error in any field. The error rate was calculated as follows: the proportion of the number of data entries containing an error on the total number of checked data entries and was estimated at 0.073 in the Kakila database.

### Step description

the structure of the Kakila database was based on the original structures of the datasets and on the functional dependencies between the data. New fields of the Kakila database were defined and approved by the data providers. Then a data dictionary was defined (Table 3). The aim of this dictionary was to produce a precise definition or description of each of the fields, based on validated scientific frameworks. The data dictionary is essential to guarantee the reusability of the database. In particular, the data dictionary ensures a clear definition of fields and limits input errors for future data entry.

**Figure 4.**
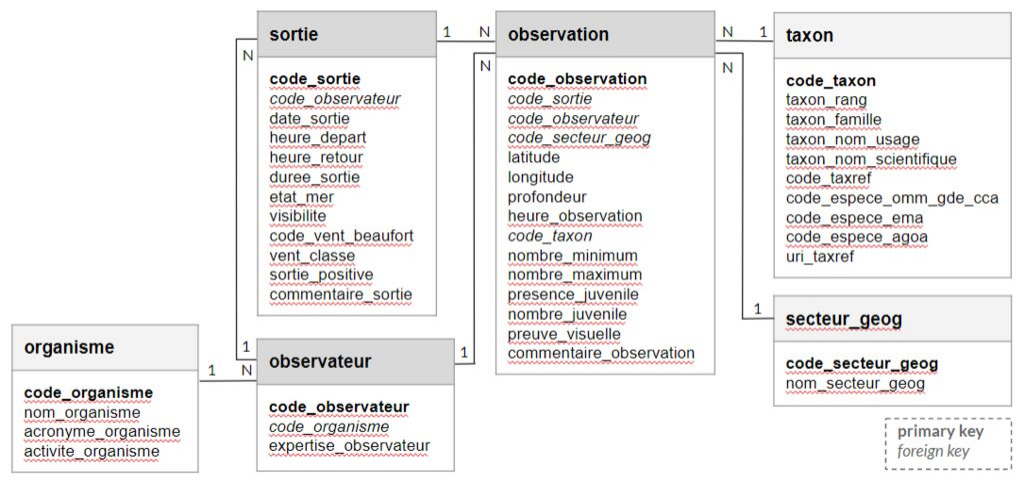
Overall structure of the Kakila database, based on six tables (observateur, organisme, sortie, observation, taxon, secteur _geog; see text for translation and description of each term).

The overall structure of the Kakila database was then designed to allow the establishment of relationships between the variables within the database. Kakila contains six main tables (Fig. 4):

- The table “observateur” (observer) lists the volunteers and whale watchers who made the observations, together with a level of expertise (from beginner "débutant" to expert "expert") for each of them.

- The table "organisme" (organisation) lists the data providers, NPOs and whale watchers.

- The table "sortie" (field trip) lists the field trips recorded in the Kakila database (n = 3249), and contains information on the date and duration of trips, observer(s) on board, sea state and visibility.

- The table "observation" (observation) lists the observations of marine mammal species recorded during the corresponding field trip. Place and time of the observation are recorded, as well as the taxon identified (see table "code_taxon") and the number of individuals observed. The availability of a picture for the observation is stated.

- The table "taxon" (taxa) lists the marine mammal taxa recorded (e.g. species, genus, family ...), including scientific and common names, as well as the TAXREF code.

- The table "secteur_geog" (geographical place) lists the geographical area that observers used to localise their observation in preference to GPS data. The geographical areas were defined using the initials of the name of the closest town or locality on the sea coast and the direction between the observation site and the locality.

The relationship of the six tables is defined by the primary/foreign key fields “code_observateur” (present in tables "observateur" and "sortie"), “code_sortie” (in tables "sortie" and "observation"), “code_taxon” (in tables "observation" and "taxon"), "code_organisme" (in tables "organisme" and "observateur") and "code_secteur_geog" (in tables "observation" and "secteur_geog") (Fig. 4).

## Geographic coverage

### Description

Our study focuses on the coastal waters surrounding the Guadeloupean Archipelago (Fig. 1). Guadeloupe is a French Island located in the West Indies. It is part of the Agoa Sanctuary, which corresponds to the French Exclusive Economic Zone of the West Indies. All observations were recorded from boats, during trips close to the coast (the most distant observation from the coast was located 35 miles (ca. 55 km) off the Island of Marie Galante).

## Taxonomic coverage

### Description

The observation consisted, whenever possible, in a taxonomic identification at the species level. Twenty-one species of cetaceans have been observed and identified. Some observations did not allow us to identify the species; in these cases, the identification was done at the family level or at the suborder level (Table 2).

### Taxa included

**Table:** 

Rank	Scientific Name	Common Name
infraorder	Cetacea	Cetaceans

## Temporal coverage

**Living time period:** 2000-2020.

### Notes

Data came from different observation structures, each with its own period of time. Data were collected between 2012-2019 for OMMAG, in 2019 for Cetacés Caraibes, between 2017 and 2019 for GED, between 2012 and 2016 for Aventures Marines, between 2007 and 2011 for BREACH, between 2012-2016 for Agoa and in 2000 for the IFAW survey.

## Usage licence

### Usage licence

Other

### IP rights notes

Data are shared under a CC-BY 4.0 licence

## Data resources

### Data package title

Kakila Dataset

### Resource link

https://data.pndb.fr/view/doi:10.48502/8bb5-pk85

### Number of data sets

6

### Data set 1.

#### Data set name

sortie

#### Data format

TSV

#### Number of columns

12

#### Download URL

https://pndb.fr/metacat/d1/mn/v2/object/urn%3Auuid%3A20deaf62-b7b7-4595-92b6-8ee627f855a5

#### Description

Content of BDD_Kakila_v2_20210221_sortie.tsv

**Data set 1.** 

Column label	Column description
code_sortie	Code of the boat trip carried out by an organisation and reported by an observer.
date_sortie	Date of the trip.
code_observateur	Observer Code.
heure_depart	Departure time of the trip.
heure_retour	Return time of the trip.
duree_sortie	Duration of the trip.
etat_mer	Sea state. Parameter value estimated by the observer using the Douglas Scale.
visibilite	Horizontal visibility. Category specifying the maximum distance at which an observer can see and identify an object located close to the horizontal plane on which he is himself (good - average - bad).
code_vent_beaufort	Wind force estimated by the observer using the Beaufort Scale from 0 to 12 (value or interval).
vent_classe	Wind force estimated by the observer classified in 4 classes (no-wind – light wind – moderate wind – strong wind).
sortie_positive	Code 1 if at least one marine mammal was observed and 0 if none was observed during the trip.
commentaire_sortie	Comments or notes about the Event.

### Data set 2.

#### Data set name

observation

#### Data format

TSV

#### Number of columns

15

#### Download URL

https://pndb.fr/metacat/d1/mn/v2/object/urn%3Auuid%3A3d06c0ef-fd9e-4b60-a54e-84b197fba3d6

#### Description

Content of BDD_Kakila_v2_20210221_observation.tsv

**Data set 2.** 

Column label	Column description
code_observation	Observation code combining the code_sortie and an observation number.
code_sortie	Code of the boat trip carried out by an organisation and reported by an observer.
code_observateur	Observer Code.
code_secteur_geog	Code of the observation site as the initials of the location (city, bay, ...) closest to the observation.
latitude	Latitude of the observation expressed in decimal degrees.
longitude	Longitude of the observation expressed in decimal degrees.
profondeur	Sea depth at the place of the observation expressed in metres from the surface. It was estimated either from a GPS sonar from the boat or by a calculation from the digital terrain model of the French Antilles available on shom.fr (source: SHOM, France). The method is specified in the comment field.
heure_observation	Observation time.
code_taxon	Internal code assigned to the taxon identified.
nombre_minimum	Observer's estimation of the minimum number of individuals observed (can be equal to nombre_maximum if the number of individuals has been precisely determined).
nombre_maximum	Observer's estimation of the maximum number of individuals observed (can be equal to nombre_minimum if the number of individuals has been precisely determined).
presence_juvenile	Presence (1) or absence (0) of juveniles at the time of observation.
nombre_juvenile	Observer’s estimation of the number of juveniles (to be completed only if presence_juvenile = 1).
preuve_visuelle	Visual evidence of observation (photography) (1) or lack of visual evidence (0). This is particularly important in the case of observers described as "beginners".
commentaire_observation	Miscellaneous comments made by the observer on the observation.

### Data set 3.

#### Data set name

organisme

#### Data format

TSV

#### Number of columns

4

#### Download URL

https://pndb.fr/metacat/d1/mn/v2/object/urn%3Auuid%3Aca9ba28a-0705-44cc-9095-24f9be3c4a7f

#### Description

Content of BDD_Kakila_v2_20210221_organisme.tsv

**Data set 3.** 

Column label	Column description
code_organisme	Code of the organisation having carried out the trip.
nom_organisme	Name of the organisation responsible for the management of reported observation data.
acronyme_organisme	Acronym of the organisation.
activite_organisme	Type of activities carried out by the organisation.

### Data set 4.

#### Data set name

secteur_geog

#### Data format

TSV

#### Number of columns

2

#### Download URL

https://pndb.fr/metacat/d1/mn/v2/object/urn%3Auuid%3A86c87a51-55a6-44de-8728-8da12072667d

#### Description

Content of BDD_Kakila_v2_20210221_secteur_geog.tsv

**Data set 4.** 

Column label	Column description
code_secteur_geog	Code of the observation site as the initials of the location (city, bay, ...) closest to the observation.
nom_secteur_geog	Name of the observation site as the name of the location (city, bay, ...) closest to the observation.

### Data set 5.

#### Data set name

observateur

#### Data format

TSV

#### Number of columns

3

#### Download URL

https://pndb.fr/metacat/d1/mn/v2/object/urn%3Auuid%3Af1f52804-d69b-4bef-a832-bedcfbeec5f5

#### Description

Content of BDD_Kakila_v2_20210221_observateur.tsv

**Data set 5.** 

Column label	Column description
code_observateur	Observer Code.
code_organisme	Code of the organisation having carried out the trip.
expertise_observateur	Level of expertise of the observer (beginner, intermediate, expert). The level of expertise is determined on the basis of the number of years of experience with regard to the identification of cetaceans.

### Data set 6.

#### Data set name

taxon

#### Data format

TSV

#### Number of columns

10

#### Download URL

https://pndb.fr/metacat/d1/mn/v2/object/urn%3Auuid%3Ab0f93874-8557-4daa-942f-af70cea9652c

#### Description

Content of BDD_Kakila_v2_20210221_taxon.tsv

**Data set 6.** 

Column label	Column description
code_taxon	Internal code assigned to the taxon identified.
taxon_rang	Taxonomic rank of the taxon identified.
taxon_famille	Family of the taxon observed.
taxon_nom_usage	Common name of the taxon identified.
taxon_nom_scientifique	Scientific name of the taxon identified in the form "genus species".
code_taxref	Code CD_REF of the taxonomic base TAXREF v.14.0 (2020-12-15).
code_espece_omm_gde_cca	Internal code used by the different observation bodies (OMMAG, Guadeloupe Evasion Découverte, Cétacés Caraïbes) to describe the species observed.
code_espece_ema	Internal code used by Aventures Marines Company to describe the species observed.
code_espece_agoa	Internal code used by the Agoa Sanctuary to describe the species observed.
uri_taxref	URI designating the taxon on the INPN site composed of a fixed URL " https://inpn.mnhn.fr/espece/cd_nom/ " followed by the TAXREF code.

## Additional information

### Discussion and foresight

Threats that cetacean populations face are multiple, but well-documented (Bedriñana-Romano et al. 2021, Campana et al. 2015, David et al. 2011, de Stephanis et al. 2013, Garcia-Cegarra et al. 2021, Gero and Whitehead 2016, Huntington 2009, Jepson et al. 2016, Jung and Madon In press, Lusseau et al. 2009, Sèbe et al. 2019, Van Waerebeek and Leaper 2008). Citizen science can play an important role in the acquisition of ecological data (e.g. Harvey et al. 2018). This is especially true for the marine megafauna, whose observation and species identification require a huge amount of time spent at sea by researchers and marine biologists, for performing accurate identifications. Large-scale scientific surveys dedicated to the study of marine mammals have proved to deliver valuable information, for example, the SCANS or the REMMOA surveys (Laran et al. 2019, SCANS-II 2008, Van Canneyt et al. 2009). However, the financial costs of such scientific surveys prevent their organisation at a sufficient interval of time required to complete and optimise the list of species, to identify fine-scale trends and to take into account mobile species not present throughout the year. Recurrent monitoring of marine mammal populations over long-time periods can only be supported by permanently present local stakeholders, such as NPOs and professionals, i.e. whale watchers. In the Guadeloupe Archipelago, local stakeholders play a major role in recording the presence of and monitoring local marine mammal populations (e.g. Gandilhon 2012, Heenehan et al. 2019, Kennedy et al. 2014, Mon école ma baleine 2019, Rinaldi 2016, Rinaldi et al. 2006, Stevick et al. 2016, Stevick et al. 2018). NPOs and whale watchers have a unique knowledge and they already collaborate on scientific studies focused on specific species (Barragán-Barrera et al. 2019, Gandilhon 2012, Stevick et al. 2016). The Kakila project aimed at taking a step further by gathering all local knowledge into a single database. This was only made possible with the involvement of all data owners in the development of the database. The process was based on a long-term collaboration between the NPO OMMAG and scientist co-authors of this paper. This allowed us to undertake a mapping of the local stakeholders, experts in the field and who may be interested in the project. They were then approached by the scientists to explain the long-term goals of the initiative. The engagement process focused on ensuring equitable contributions and mitigating any tensions related to the use of the data. Once agreements and data were provided, the project undertook the delicate phase of data curation, harmonisation, standardisation and development of the database architecture. Each collector had his/her own tabulated file for entering observations with no central data store and access interface. However, all these datasets share common variables that constituted the common basis for the Kakila database construction. Data owners were involved in this technical process and their feedback was requested and taken into account (e.g. naming fields) to foster a sense of ownership and ensure the long-term usage of the database.

Providing metadata has been eased by a development version of MetaShARK. Since this application was maturing, some parts of the data description had to be handled manually: turning the files encoding from Windows-1252 to UTF-8 and correcting EML Assembly Line templates when needed.

The Kakila database is the first attempt at gathering all available local knowledge on cetacean presence in the Guadeloupe Archipelago. Clearly the long-term strategy to maintain and enrich the Kakila database must focus on careful monitoring of stakeholders' interests, motivations and ultimate expectations. One of its first scientific valorisations will be to help detect and identify key areas of interaction between cetaceans and marine traffic in the Guadeloupe Archipelago in the framework of the TRAFIC project *^2^. In addition, we hope to be able to develop such a database for other small island countries and territories of the Greater Caribbean Area.

## Figures and Tables

**Figure 2a.**
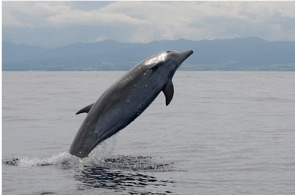
Bottlenose dolphin (*Tursiops truncatus*)

**Figure 2b.**
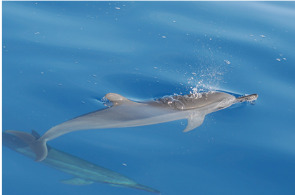
Pantropical spotted dolphin (*Stenella attenuata*)

**Figure 2c.**
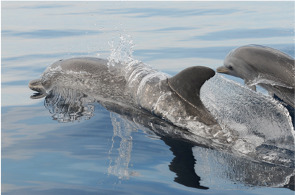
Atlantic spotted dolphin (*Stenella frontalis*)

**Figure 2d.**
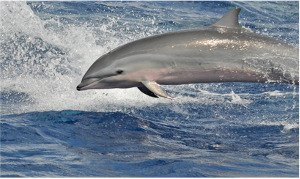
Fraser’s dolphin *(Lagenodelphis hosei*).

**Figure 3a.**
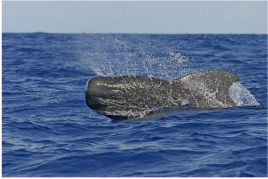
Short-finned pilot whale (*Globicephala macrorhynchus*)

**Figure 3b.**
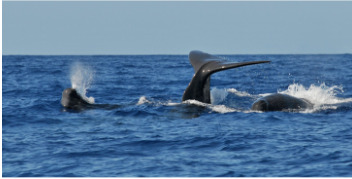
Sperm whale (*Physeter macrocephalus*)

**Figure 3c.**
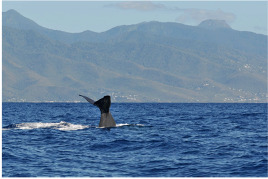
Sperm whale (*Physeter macrocephalus*)

**Figure 3d.**
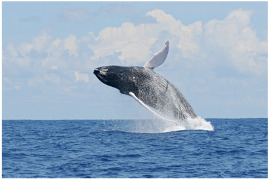
Humpback whale (*Megaptera novaeangliae*).

**Table 1.** Description of the FAIRisation process of the Kakila database.

**FAIR principles (Wilkinson et al. 2016)**	**FAIRness assessment criteria used for the Kakila database**
FINDABLE	- Using unique identifiers for each observation occurrence, observer, boat excursion, taxon, collector organism and geographic sectors. - Making persistent metadata and datasets thanks to the deposit to the French Pôle National de données de Biodiversité (PNDB, https://www.pndb.fr/) which is a national infrastructure data repository. - Providing a data dictionary to guarantee the reusability of the database. - Using the Ecological Metadata Language (EML) internationally recognised standard to describe the database metadata and its associated projects, including standardised search keywords. - Using a metadata format validator thanks to the MetaShARK (Arnaud et al. 2020). - Using a versioning system to allow future updates. - Generating a Darwin Core Archive from the Kakila database. The Darwin Core Standard (DwC) offers a stable, straightforward and flexible framework for compiling biodiversity data, notably occurrences, from varied and variable sources (Wieczorek et al. 2012).
ACCESSIBLE	- Storing data in the PNDB repository with respect to the guidelines for quality standards (e.g. use of EML). - Efficient and rich services for various uses and users provided by the PNDB. - Working to adapt the Kakila database in order to integrate it in the GBIF.
INTEROPERABLE	- Using standard vocabularies for some fields (e.g. Beaufort Wind Scale for the wind speed). - Using keywords of international thesaurus, such as GEMET/INSPIRE (GEMET 2008) and AGROVOC (Food and Agriculture Organization of the United Nations 1980). - Using a data dictionary including the Darwin Core mapping. - Associating a Darwin Core archive with the Kakila database. The Darwin Core Standard (DwC) offers a stable, straightforward and flexible framework for compiling biodiversity data from varied and variable sources (Wieczorek et al. 2012).
REUSABLE	- Using an open format for the dataset (Tab Separated Values .tsv and OpenDocument .ods for the original database) and open source software to reuse it. - Including in the EML metadata the provenance for raw and derived data. - Explaining in this data paper the data processing steps, the data curation protocol, the data quality assurance processes, the methods and tools that permit long term integrity and understandability of data. - Using a time range clearly mentioned in the EML metadata and in this data paper. The same applies for geographical and taxonomic coverages and the CC-BY licence and rules for large reuse. - Using a Darwin Core Archive to facilitate the reusability of the Kakila database, because it enables the publication into the GBIF. This compact package (a ZIP file) contains interconnected text files and enables users to share their data using common terminology.

**Table 2.** List of taxa recorded between 2000 and 2020 from the Guadeloupean Archipelago.

Rank of the taxa identified	Family	Scientific name	Common name (in French)	Common name (in English)	code_taxref
Infraorder		Cetacea	Cétacés	Cetaceans	186224
Family	Balaenopteridae	Balaenopteridae	Balénoptéridés - rorquals	Rorquals	186226
	Delphinidae	Delphinidae	Delphinidés	Oceanic dolphins	186227
	Kogiidae	Kogiidae	Kogiidés - petits cachalots	Kogidae	351415
	Physeteridae	Physeteridae	Physétéridés - cachalots	Sperm whales	186231
	Ziphiidae	Ziphiidae	Ziphiidés - Hyperoodontidés	Beaked whales	186232
Species	Balaenopteridae	*Balaenoptera acutorostrata*	Petit Rorqual	Minke whale	60856
		*Balaenoptera physalus*	Rorqual commun	Fin whale	60861
		*Megaptera novaeangliae*	Baleine à bosse	Humpback whale	60867
		*Balaenoptera edeni*	Rorqual de Bryde	Bryde’s whale	60860
	Delphinidae	*Feresa attenuata*	Orque naine ou pygmée	Pygmy killer whale	60883
		*Globicephala macrorhynchus*	Globicéphale tropical	Short-finned pilot whale	60887
		*Lagenodelphis hosei*	Dauphin de Fraser	Fraser’s dolphin	60897
		*Orcinus orca*	Orque Epaulard	Killer whale, Orca	60905
		*Peponocephala electra*	Péponocéphale ou Dauphin d'Electre	Melon-headed whale, Electra dolphin	60908
		*Pseudorca crassidens*	Pseudorque	False killer whale	60911
		*Stenella coeruleoalba*	Dauphin bleu et blanc	Striped dolphin	60918
		*Stenella attenuata*	Dauphin tacheté pantropical	Pantropical spotted dolphin	60914
		*Stenella clymene*	Dauphin de Clymène	Clymene dolphin	60917
		*Stenella frontalis*	Dauphin tacheté de l'Atlantique	Atlantic spotted dolphin	60921
		*Stenella longirostris*	Dauphin à long bec	Spinner dolphin	60916
		*Steno bredanensis*	Steno rostré	Rough-toothed dolphin	60924
		*Tursiops truncatus*	Grand dauphin	Bottlenose dolphin	60927
	Kogiidae	*Kogia sima*	Cachalot nain	Dwarf sperm whale	79307
	Physeteridae	*Physeter macrocephalus*	Grand cachalot	Sperm whale	60949
	Ziphiidae	*Mesoplodon europeaus*	Baleine à bec de Gervais	Gervais’ beaked whale	60962
		*Ziphius cavirostris*	Baleine à bec de Cuvier	Cuvier’s beaked whale	60970

**Table 3.** Data dictionary - metadata repository - of the Kakila DB. Datasets and Column labels are also presented in the "Data resources" part. The Darwin core data standards are described in Wieczorek et al. (2012).

**Datasets and Column labels**	**Definition**	**Data type**	**Darwin Core term code**	**Darwin Core term definition**
**Dataset "sortie" (Trip)**				
**code_sortie**	**Code of the boat trip carried out by an organisation and reported by an observer**	**Text**	**eventID**	**An identifier for the set of information associated with an Event (something that occurs at a place and time). May be a global unique identifier or an identifier specific to the data set.**
date_sortie	Date of the trip.	Date	eventDate	The date-time or interval during which an Event occurred. For occurrences, this is the date-time when the event was recorded.
**code_observateur**	**Observer Code**	**Text**		
heure_depart	Departure time of the trip.	Hour		
heure_retour	Return time of the trip.	Hour		
duree_sortie	Duration of the trip.	Numeric		
etat_mer	Sea state. Parameter value estimated by the observer using the Douglas Scale.	Text	fieldNotes	One of a) an indicator of the existence of, b) a reference to (publication, URI), or c) the text of notes taken in the field about the Event.
visibilite	Horizontal visibility. Category specifying the maximum distance at which an observer can see and identify an object located close to the horizontal plane on which he is himself (good - average - bad).	Text		
code_vent_beaufort	Wind force estimated by the observer using the Beaufort Scale from 0 to 12 (value or interval).	Numeric		
vent_classe	Wind force estimated by the observer classified in 4 classes (no-wind – light wind – moderate wind – strong wind).	Text		
sortie_positive	Code 1 if at least one marine mammal was observed and 0 if none was observed during the trip.	Numeric		
commentaire_sortie	Miscellaneous comment associated with the boat trip.	Text	eventRemarks	Comments or notes about the Event.
**Dataset "observateur" (observer)**				
**code_observateur**	**Observer Code**	**Text**		
**code_organisme**	**Code of the organisation having carried out the trip**	**Text**		
expertise_observateur	Level of expertise of the observer (beginner, intermediate, expert). The level of expertise is determined on the basis of the number of years of experience with regard to the identification of cetaceans.	Text	identificationRemarks	Comments or notes about the Identification.
**Dataset "observation" (observation)**				
**code_observation**	**Observation code combining the code_sortie and an observation number**	**Text**	**occurrenceID**	**An identifier for the Occurrence (as opposed to a particular digital record of the occurrence). In the absence of a persistent global unique identifier, construct one from a combination of identifiers in the record that will most closely make the occurrenceID globally unique.**
**code_sortie**	**Code of the boat trip carried out by an organisation and reported by an observer**	**Text**	**eventID**	**An identifier for the set of information associated with an Event (something that occurs at a place and time). May be a global unique identifier or an identifier specific to the data set.**
**code_observateur**	**Observer Code**	**Text**		
**code_secteur_geog**	**Code of the observation site as the initials of the location (city, bay, ...) closest to the observation**	**Text**		
latitude	Latitude of the observation expressed in decimal degrees.	Numeric	decimalLatitude	Geographic Longitude (in decimal degree, using the spatial reference system in "Reference system")
longitude	Longitude of the observation expressed in decimal degrees.	Numeric	decimalLongitude	Geographic Latitude (in decimal degree, using the spatial reference system in "Reference system")
profondeur	Sea depth at the place of the observation expressed in metres from the surface. It was estimated either from a GPS sonar from the boat or by a calculation from the digital terrain model of the French Antilles available on shom.fr (source: SHOM, France). The method is specified in the comment field.	Numeric	minimumDepthInMetres	The lesser depth of a range of depth below the local surface, in meters.
heure_observation	Observation time.	Hour	eventTime	The time or interval during which an Event occurred.
**code_taxon**	**Internal code assigned to the taxon identified**	**Text**		
nombre_minimum	Observer's estimation of the minimum number of individuals observed (can be equal to nombre_maximum if the number of individuals has been precisely determined).	Numeric	individualCount	The number of individuals represented present at the time of the Occurrence.
nombre_maximum	Observer's estimation of the maximum number of individuals observed (can be equal to nombre_minimum if the number of individuals has been precisely determined).	Numeric		
presence_juvenile	Presence (1) or absence (0) of juveniles at the time of observation.	Numeric	occurrenceRemarks	Comments or notes about the Occurrence.
nombre_juvenile	Observer’s estimation of the number of juveniles (to be completed only if presence_juvenile = 1).	Numeric	occurrenceRemarks	Comments or notes about the Occurrence.
preuve_visuelle	Visual evidence of observation (photography) (1) or lack of visual evidence (0). This is particularly important in the case of observers described as "beginners".	Numeric		
commentaire_observation	Miscellaneous comments made by the observer on the observation.	Text	occurrenceRemarks	Comments or notes about the Occurrence.
**Dataset "organisme" (organisation)**				
**code_organisme**	**Code of the organisation having carried out the trip**	**Text**		
nom_organisme	Name of the organisation responsible for the management of reported observation data.	Text	recordedBy	A list (concatenated and separated) of names of people, groups, or organizations responsible for recording the original Occurrence. The primary collector or observer, especially one who applies a personal identifier (recordNumber), should be listed first.
acronyme_organisme	Acronym of the organisation.	Text	ownerInstitutionCode	The name (or acronym) in use by the institution having ownership of the object(s) or information referred to in the record.
activite_organisme	Type of activities carried out by the organisation.	Text		
**Dataset "secteur_geog" (observation site)**				
**code_secteur_geog**	**Code of the observation site as the initials of the location (city, bay, ...) closest to the observation**	**Text**		
nom_secteur_geog	Name of the observation site as the name of the location (city, bay, ...) closest to the observation.	Text	locationID	An identifier for the set of location information (data associated with dcterms:Location). May be a global unique identifier or an identifier specific to the data set.
**Dataset "taxon" (taxon)**				
**code_taxon**	**Internal code assigned to the taxon identified**	**Text**		
taxon_rang	Taxonomic rank of the taxon identified.	Text	taxonRank	Taxonomic rank of the taxon identified, using the Taxonomic Rank GBIF Vocabulary
taxon_famille	Family of the taxon observed.	Text	family	The full scientific name of the family in which the taxon is classified.
taxon_nom_usage	Common name of the taxon identified.	Text	originalNameUsage	The taxon name, with authorship and date information if known, as it originally appeared when first established under the rules of the associated nomenclaturalCode. The basionym (botany) or basonym (bacteriology) of the scientificName or the senior/earlier homonym for replaced names.
taxon_nom_scientifique	Scientific name of the taxon identified in the form "genus species".	Text	scientificName	The full scientific name, with authorship and date information if known. When forming part of an Identification, this should be the name in lowest level taxonomic rank that can be determined. This term should not contain identification qualifications, which should instead be supplied in the IdentificationQualifier term.
code_taxref	Code CD_REF of the taxonomic base TAXREF v.14.0 (2020-12-15).	Numeric		
code_espece_omm_gde_cca	Internal code used by the different observation bodies (OMMAG, Guadeloupe Evasion Découverte, Cétacés Caraïbes) to describe the species observed.	Text		
code_espece_ema	Internal code used by Aventures Marines Company to describe the species observed.	Text		
code_espece_agoa	Internal code used by the Agoa Sanctuary to describe the species observed.	Text		
uri_taxref	URI designating the taxon on the INPN site composed of a fixed URL "https://inpn.mnhn.fr/espece/cd_nom/" followed by the TAXREF code	Text	taxonID	An identifier for the set of taxon information (data associated with the Taxon class). May be a global unique identifier or an identifier specific to the data set.

## Footnotes

*1 See for instance: https://www.unenvironment.org/explore-topics/oceans-seas/what-we-do/working-regional-seas/coastal-zone-management or https://www.un.org/sustainabledevelopment/wp-content/uploads/2017/05/Ocean-fact-sheet-package.pdf

*2 See also the OHM Littoral Caraïbe webpage: https://ohm-littoral-caraibe.in2p3.fr/methodologie#trafic
